# Autologous HIV-1 Clade-B Nef Peptides Elicit Increased Frequency, Breadth and Function of CD8^+^ T-Cells Compared to Consensus Peptides

**DOI:** 10.1371/journal.pone.0049562

**Published:** 2012-11-19

**Authors:** Mehrnoosh Doroudchi, Oleg Yegorov, Tom Baumgartner, Anne-Elen Kernaleguen, Gaelle Breton, Michel L. Ndongala, Mohamed-Rachid Boulassel, Jean-Pierre Routy, Nicole F. Bernard, Rafick-Pierre Sékaly, Bader Yassine-Diab

**Affiliations:** 1 Laboratoire d'Immunologie, Centre de Recherche du Centre Hospitalier de l'Université de Montréal (CR-CHUM) Saint-Luc, Montréal, Québec, Canada; 2 Laboratoire d'Immunologie, Département de Microbiologie et d'Immunologie, Université de Montréal, Québec, Canada; 3 INSERM U743, CR-CHUM, Université de Montréal, Montréal, Québec, Canada; 4 Department of Immunology, School of Medicine, Shiraz University of Medical Sciences, Shiraz, Iran; 5 Vaccine and Gene Therapy Institute-Florida, Port St Lucie, Florida, United States of America; 6 Department of Experimental Medicine, McGill University, Montréal, Québec, Canada; 7 Immunodeficiency Service and Division of Hematology, Royal Victoria Hospital, McGill University Health Centre, McGill University, Montréal, Québec, Canada; 8 Caprion/ImmuneCarta Services, Montréal, Québec, Canada; MRC National Institute for Medical Research, United Kingdom

## Abstract

**Objective:**

To determine the function and phenotype of CD8^+^ T-cells targeting consensus and autologous sequences of entire HIV-1 Nef protein.

**Methods:**

Multiparameter flow cytometry-based analysis was used to evaluate the responses of two treatment naïve HIV-infected individuals, during primary and the chronic phases of infection.

**Results:**

A greater breadth and magnitude of CD8 IFN-γ responses to autologous compared to clade-B consensus peptides was observed in both subjects. Cross recognition between autologous and consensus peptides decreased in both subjects during progression from primary to chronic infection. The frequencies of TEMRA and TEM CD8^+^ T-cells targeting autologous peptides were higher than those targeting consensus peptides and were more polyfunctional (IFN-γ^+^ Gr-B^+^ CD107a^+^).

**Conclusions:**

Our data indicate superior sensitivity and specificity of autologous peptides. The functional and maturational aspects of “real” versus “cross-recognized” responses were also found to differ, highlighting the importance of a sequence-specific approach towards understanding HIV immune response.

## Introduction

A better understanding of the interplay between HIV and the immune system is necessary for the development of a safe and effective HIV vaccine. To date, we have gained a comprehensive understanding of HIV proteins and epitopes targeted by the immune system, as well as the mechanisms of immune evasion by the virus [1]–[3]. Although CD8^+^ T cell response target all HIV-1 proteins during infection, the relative contribution of each protein to viral evolution varies during acute and chronic phases [4]. Gag and Nef proteins contain the highest density of epitopes recognized by T-cell responses [4]–[6] and HIV-1 Nef, an early viral regulatory protein, is the dominant CD8 T-cell targeted protein during primary HIV infection, while responses to HIV-1 Gag dominate the CD8 T-cell response during the chronic phase [4]. This is not unexpected given that HIV-1 Nef is expressed early in the viral replicative cycle [6] and contains a large number of CTL epitopes [4], [7]. The relative contribution of Nef-specific CD8 T-cell responses to the total HIV-induced CD8 T-cell response, however, decreases early after the HIV infection [8].

Nef protein induces its own secretion in bioactive micro vesicles by infected cells [9], [10], or is transferred to bystander cells through cell-cell contact [11]. It is well established that HIV proteins, including Nef, are subject to high rates of mutation as a result of high viral replication and the error prone HIV reverse transcriptase [12]. The large degree of variation of the HIV proteome is one of the major obstacles for the development of an effective HIV vaccine [13]. The rapid evolution of HIV variants within an infected individual results in the appearance of escape mutants. At the population level, this phenomenon results in diversification of viral sequences [14]. To overcome this complexity for identifying Nef-specific responses, strain-specific laboratory isolates of HIV-1 [15], M-group consensus viral sequences [13] have been used to measure immune responses. This approach has been adopted for practicality and cost-efficiency, and has produced a large body of information about the immune response directed against the virus. The accuracy and reliability of the experimental data using this approach to identify in vivo responses however is poorly defined. Information concerning the correlation between the breadth, magnitude, phenotype, and function of responses induced by autologous versus consensus proteins of the virus is also lacking. In a previous study, Altfed *et al.* reported a higher magnitude and breadth of responses to pools of autologous HIV-1 peptides compared to consensus clade-B peptides [16]. In addition, we observed better proliferation of Nef-specific CD8^+^ T-cells in response to dendritic cells (DC) electroporated with autologous versus consensus viral RNA. However, CD8 T-cell differentiation, maturation and effector function stimulated in *ex-vivo* PBMC cultures with consensus peptides versus autologous wild type virus and mutant virus is unknown.

In this study, we compared CD8^+^ T-cell IFN-γ responses to autologous and consensus B Nef peptides in treatment naïve viremic subjects during both the primary and chronic phase of infection, with the aim to better define their maturational and functional state.

## Methods

### Ethics statement

Archived peripheral blood mononuclear cells (PBMC) samples from (n = 2) HIV-infected subjects were used. This study received approval from the Institution Review Board of the McGill University Health Center and CHUM-Research Center, and was conducted in compliance with the principles included in the Declaration of Helsinki. Both patients provided written informed consent for their participation to the study.

### Subjects

Untreated viremic HIV-infected Caucasian individuals were recruited to this study from the Montreal HIV Primary Infection (PI) Cohort in the primary phase of infection and followed longitudinally. To minimize the effect of treatment and viral load on the study results, untreated viremic subjects with similar viral loads (VL) during the primary phase of infection were chosen, thereby limiting the number of subjects available for the study (n = 2). Both subjects were followed for two years, during which viral load (VL), total CD4, and CD8 T-cell counts were measured on a monthly or bimonthly basis as described elsewhere [17]. PBMC from leukapheresis blood samples available from two time points post infection (day 92 and day 448 (#039) and day 155 and day 372 (#016)) were used for the majority of the studies reported here. The approximate date of infection was estimated according to the guidelines proposed by the Acute HIV Infection and Early Disease Research Program sponsored by the National Institutes of Allergy and Infectious Disease, Division of AIDS (Bethesda, Maryland) [18]. In addition, information obtained from questionnaires addressing the time of high-risk behavior for HIV transmission was taken into account in assigning a date of infection, when consistent with biological tests. The results of a less sensitive HIV-1 antibody enzyme immunoassay (LS-EIA) which identifies infected subjects within a window period of 170 days from infection (95% confidence interval 162–183 days) were also used to confirm the estimated date of infection [19]. Both individual's HLA class I type was determined as described previously [20]. Briefly, genomic DNA was extracted from PBMC or EBV-transformed cells using a QIAamp DNA blood kit (Qiagen, Mississauga, Ontario, Canada). Both subjects were typed for HLA class I alleles by sequence-based typing using kits from Atria Genetics (South San Francisco, CA).

### Viral RNA isolation and sequencing

**Figure 1:**
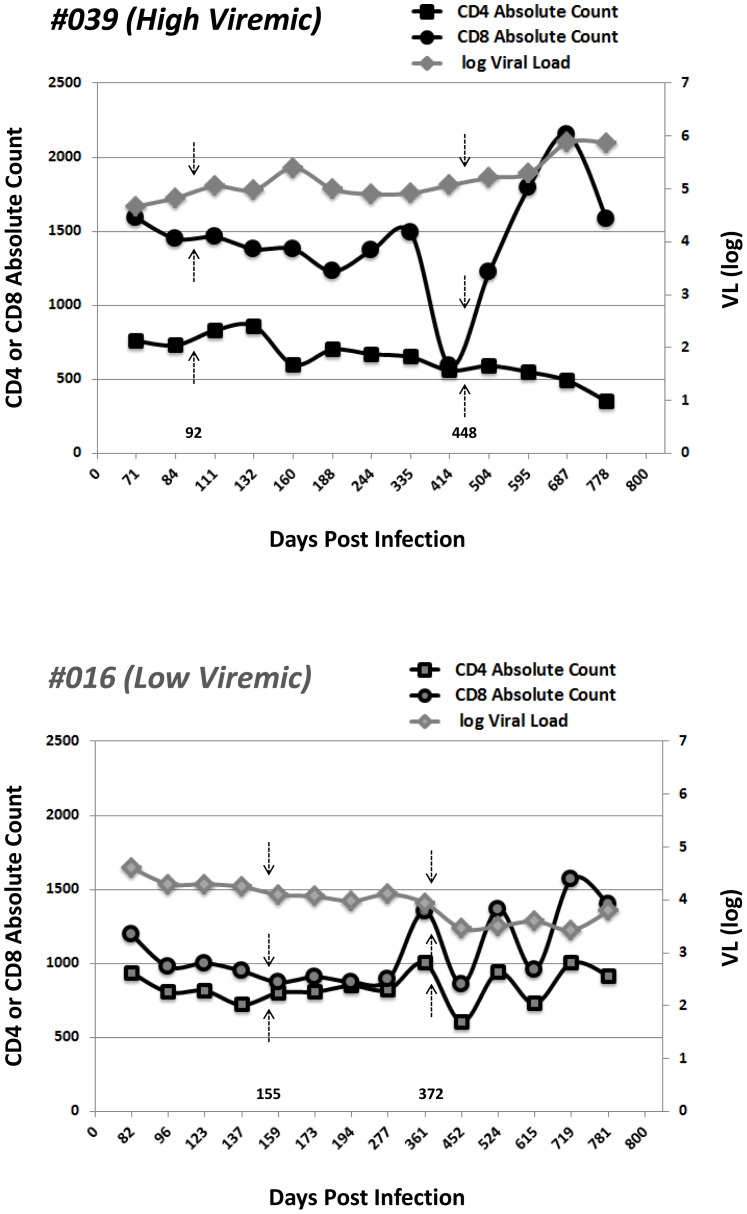
Viral load, CD4+ and CD8^+^ T-cell counts (n = 2). Viral load (triangles; 46731 and 41023 viral RNA copy/ml of plasma), absolute CD4^+^ T-cell count (squares) and absolute CD8^+^ T-cell count (circles) are shown for two time points. The grey arrows show the time points in which the virus was sequences and the black arrows indicate the time points in which the leukapheresis blood was taken and PBMCs were used in this study.

HIV-1 RNA in autologous plasma from primary and chronic time points (Figure 1) was extracted, reverse transcribed, and amplified by nested PCR. The resulting half-genome amplicons were then directly sequenced as described previously [21]. Briefly, half the genomes are amplified using specifically primed cDNA. Two amplicons per genome were directly sequenced using primers designed to highly conserved regions. Ambiguous bases were recorded using the IUB code for mixed bases (e. g. Y for C or T). Amplification reactions produce a single band of the expected molecular weight. PCR products were purified for direct sequencing using an ABI 3700 automated capillary sequencer. Sequences were edited using EditView and were assembled into a single contig and then aligned using the Seqman and Megalign programs respectively (DNAstar Inc., Madison, WI). This method was used to derive autologous virus genome sequences from which to design autologous strain peptide libraries from primary and chronic replicating viruses.

### Peptides

Consensus clade-B Nef peptides (15-mer) were obtained from the National Institutes of Health AIDS Research and Reference Reagent Program (Rockville, MD, USA). Two sets of 15-mer peptides with 11 amino acid (a. a.) overlaps, corresponding to the Nef sequence of the two study subjects, were synthesized by Sigma-Aldrich PEPscreen (The Woodlands, TX, USA). The approximate purity of the peptides was 80%, as measured by HPLC and mass spectroscopy. Individual peptides were dissolved in DMSO (Sigma-Aldrich, USA) at a concentration of 50 mg/ml and stored at −80°C. Eight to ten peptides were first pooled in a matrix format [18], such that the concentration of each peptide used for stimulation was 100 ng/µl. For second round assays, individual peptides were tested at a final concentration of 500 ng/ml. For the comparison between the time points, the same set of peptides corresponding to the primary and chronic time points were included in the test design.

### CD107a mobilization and Intracellular cytokine staining assay (ICS)

Frozen PBMCs were thawed and used for ICS staining, after 16 hrs of stimulation with cognate peptide. The stimulation of 2×10^6^ PBMCs was done in 1 ml of RPMI 1640 medium, containing 10% Fetal Bovine Serum (Sigma-Aldrich, USA). The CD107a PE-Cy5 antibody was added at the time of stimulation. Cytokine secretion was stopped after 60 minutes by adding Brefeldin-A and Monensin (Sigma-Aldrich, USA) at final concentrations of 0.5 and 5 µg/ml. The stimulated cells were incubated for 15 hours at 37°C. PBMCs were washed and stained with anti-CD3 Pacific-Blue, anti-CD4 Am-Cyan, anti-CD8 Alexa-700, anti-CCR7 PE-Cy7, anti-CD45RA APC and anti-CD27 APC-Cy7 antibodies at 4°C for 20 minutes. The cells were then washed and fixed with FACS-lysing solution (BD Biosciences, Mississauga, ON, CA) for another 20 minutes at room temperature (RT). The cells were permeabilized by adding 0. 25% Saponin (Sigma-Aldrich, USA) in PBS and immediately washed and stained intracellularly with anti-IFN-γ FITC, anti-IL-2 PE, and anti-Gr-B PE-Texas-Red for 30 minutes at RT. The cells were subsequently fixed in 2% formaldehyde. Antibodies were purchased from BD Biosciences, except for anti-Gr-B PE-Texas-Red (Caltag, CA, USA). Live lymphocytes were gated by forward and side scatter. A total of 2×10^6^ events for each sample were acquired using the BD LSRII flow cytometer (BD Biosciences, Mississauga, ON, CA). The data was analyzed using DIVA software (BD Biosciences, Mississauga, ON, CA).

The low magnitude responses to peptide variants were only considered positive when the magnitude of the response to one or the other variant was above the threshold (three times greater than the background staining in the non-stimulated) for a positive response. For a response to be included in the analysis of functional subpopulations, the frequency of the subpopulation had to be greater than the value obtained from the formula below:
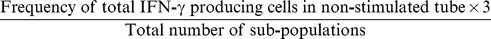



### Statistical analysis

Statistical analysis was performed using GraphPad Prism 2.0 software. In order to compare the means between peptides or time points, we used Wilcoxon signed rank test. In order to compare the means of unpaired data, we used Student t-test and Mann Whitney test. Chi-square test was used to compare the cell frequencies between groups.

## Results

### HIV-1 infected subjects, peptides and incentive

Two viremic and untreated HIV-infected subjects (#039 and #016) with comparable VL (46731 versus 41023 viral RNA copy/ml of plasma) were recruited during the primary phase of infection (day 92 and day 155 respectively) and followed longitudinally (Figure 1). A summary of the VL and T-cell counts for the two subjects is provided in Table S1. HLA haplotypes for the subjects were as follows: #039 = A*0101, A*2301, B*4901, B5801; #016 = A*1101, A*2402, B*3501, B*5701. On follow-up, viremia was shown to increase in one subject (#039) and decrease in the other (#016) subject, heretofore denoted as high viremic (>10,000 copies/ml) and low viremic (<10,000 copies/ml) subjects, respectively.

Based on the compositional matrix adjustment method (http://blast.ncbi.nlm.nih.gov/Blast.cgi), the percentage of a. a. sequence similarity of autologous HIV-1 Nef with the clade-B consensus Nef varied between 83–85% and 84–85% for the high and low viremic subjects, respectively. HIV-1 Nef proteins from the two individuals showed 77–78% identity (Figure S1). Notably, despite the high degree of a. a. conservation between clade-B consensus and autologous HIV variants, a total of 60 out of 64 and 95 out of 100 peptides were dissimilar for the low and high viremic subjects, respectively. In addition, only 5 pairs of the 164 autologous peptides generated in both subjects were similar.

### The magnitude and breadth of CD8^+^ T-cell IFN-γ responses to clade-B consensus Nef peptides underestimates the response to autologous HIV-1 Nef peptides

**Figure 2:**
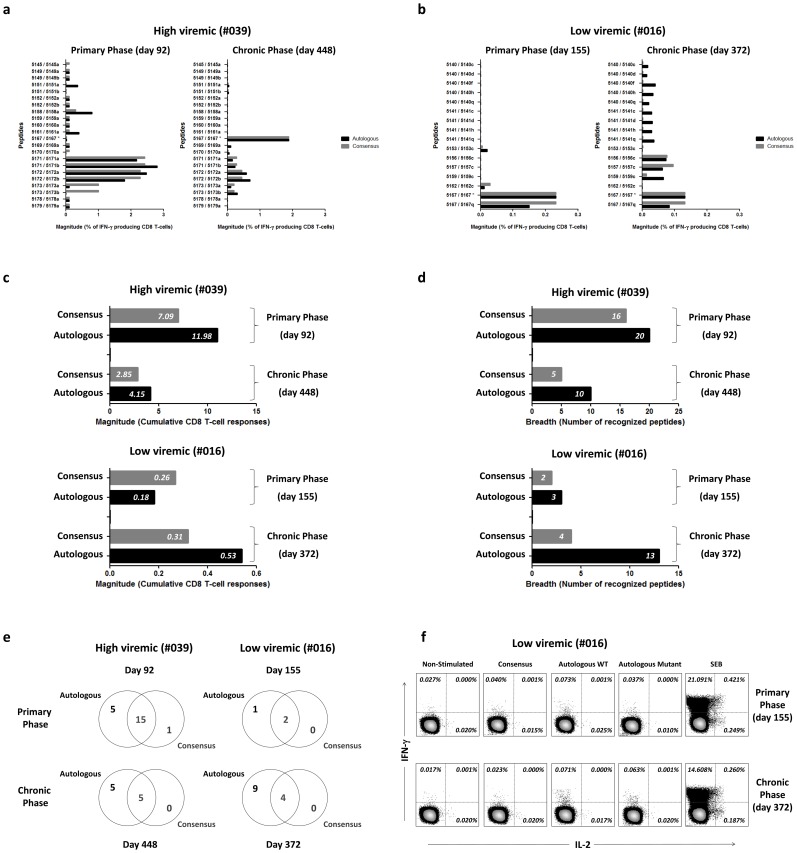
Breadth and magnitude of CD8^+^ T-cell IFN-γ responses to clade-B consensus and autologous HIV-1 Nef peptides. Frequencies and magnitudes of total Nef-specific CD8^+^ T-cell IFN-γ response to consensus and autologous HIV-Nef peptides by consensus peptides in high viremic subject (a) and low viremic subject (b). A decrease (#039) and an increase (#016) in the breadth of CD8^+^ T-cell responses to both consensus and autologous HIV-Nef sequences is shown during transition from primary (T1) to chronic (T2) infection. For high viremic subject, letter “a” in front of the peptide shows that the peptide belongs to visit 01 virus and letter “b” shows that the peptide belongs to visit 08 virus. For the low viremic subject, letters “c, d, f, and h” in front of the peptide shows that the peptide belongs to visit 01 virus and letter “q” shows that the peptide belongs to visit 10 virus. The asterisk shows that the consensus and the autologous peptides had similar a. a. sequences. CD8^+^ T-cells (%) producing IFN-γ upon stimulation with consensus and autologous HIV-Nef sequences are shown. Only high responses (>0.1 for #039 and >0.01 for #016) are shown. (c) Total magnitude of Nef-specific T-cell responses in low viremic subject (top) and high viremic subject (bottom) directed at either consensus or autologous peptides. (d) Breadth of total anti-Nef responses detected, expressed as the number of recognized peptides. (e) Number of peptides that induced CD8^+^ T-cell IFN-γ responses in either category of clade-B consensus or autologous HIV-1 Nef peptides, by phase of infection. (f) Divergent evolution of CD8^+^ IFN-γ responses to consensus HIV-Nef peptide 5140 (WSKRSVVGWPTVRER), autologous WT HIV-Nef peptide 5140d (WSKRSVPGWNIIRER) and autologous mutant HIV-Nef peptide 5140q (WSKRGVPGWNTIRER) over time. Percentages of cytokine producing subpopulations in total CD8^+^ T-cells in low viremic subject are shown: Magnitude of IFN-γ production versus the magnitude of IL-2 production (% of CD8^+^ T-cells) from PBMC following stimulation. The negative control = non-stimulated, positive control tube = (SEB) used *Staphylococcus aureus* Enterotoxin B superantigen as stimulant. Plots are gated on CD3^+^ CD8^+^ T-cells.

PBMCs from both subjects #039 and #016 were obtained following leukapheresis during the primary phase of infection (day 92 and day 155 respectively) and chronic phase of infection (day 448 and day 372 respectively) to obtain sufficient PBMCs to test the large number of peptides analyzed in this study. In the first set of experiments, we compared the breadth and magnitude of CD8^+^ T-cell responses secreting IFN-γ to consensus clade-B versus autologous Nef peptides (Table 1). The magnitude and breadth of CD8^+^ T-cell IFN-γ responses to autologous peptides in PBMC were higher than consensus peptides, independent of the phase of infection (Figures 2a and 2b, Figure S2). The difference in the magnitude of CD8^+^ T-cell IFN-γ responses to autologous versus consensus peptides was more evident in primary infection (day 92) for the high viremic subject (11.98% versus 7.09%) and in the chronic phase (day 372) for the low viremic subject (0.54% versus 0.32%), with the high and low viremic subjects demonstrating divergent responses over time (Figure 2c). Most striking, 2 of 3 primary peptide responses (consensus/autologous peptides 5153/5153c and 5162/5162c) in the subject #016 (day 155), that went on to control virus replication, disappeared in chronic infection (day 372), whereas the early response to peptide 5167 (WVYHTQGYFPDWQNY); which had identical consensus and autologous sequences in the primary phase of infection; remained throughout with slight decrease (Figure 2b). Importantly, using web-based approach to CTL epitope prediction (a combined algorithm integrating MHC class I binding, TAP transport efficiency, and proteasomal cleavage predictions: http://tools.immuneepitope.org/analyze/html/mhc_processing.html, detailed analysis of these 3 peptides showed that peptide 5167 (but not peptides 5153 and 5162), contains five different CTL epitopes, restricted by three different alleles (B*5701, B*3501, and A*2402) with high binding affinities (Table S2). However, the emergence of escape mutation within this peptide (5167q) during the chronic phase of infection (day 372) was associated with reduced peptide recognition and T cell responses. In addition, new specificities arose in the low viremic subject during chronic infection (day 372), that were detected only with autologous peptides following escape mutation. This response was not detected in the high viremic subject (#039) except for one low response that arose to the autologous peptide 5173b (LTFGWCYKLVPMEED). Numerous early responses to both autologous and consensus sequences were not detected or diminished in frequency in this subject (#039) during transition to chronic infection (day 448) whereas the immunodominant IFN-γ response to a single peptide increased in frequency (peptide 5167; which had an identical consensus and autologous sequences: WVYHTQGYFPDWQNY). Analysis of the total number of autologous peptides recognized in both subjects revealed a broader IFN-γ response, when compared to consensus peptides across time points (Figure 2d). We were unable to detect dual positive IFN-γ^+^ IL-2^+^ or IL-2^+^ Nef-specific CD8^+^ T-cell responses consistent with previous reports of decreased IL-2 production and proliferation of HIV-specific T cells associated with persistent viremia [22], [23].

**Table 1:** Comparison of peptide clade-B consensus sequence and autologous sequence (WT and mutants) corresponding to the Nef-specific CD8 T-cell responses detected in study subjects.

Clade B Nef Consensus		#039 Nef Autologous (High Viremic)				#016 Nef Autologous (Low Viremic)			
Primary & Chronic Phases		Primary Phase day 92 (WT)		Chronic Phase day 448 (Mutants)		Primary Phase day 155 (WT)		Chronic Phase day 372 (Mutants)	
*Name*	*Peptide Sequence*	*Name*	*Peptide Sequence*	*Name*	*Peptide Sequence*	*Name*	*Peptide Sequence*	*Name*	*Peptide Sequence*
5140	WSKRSVVGWPTVRER					5140c	WSKRSV**P**GW**N**T**I**RER	5140q	WSKR**G**V**P**GW**N**T**I**RER
						5140d	WSKRSV**P**GW**NII**RER		
						5140f	WSKRSV**P**GW**NAI**RER		
						5140h	WSKRSV**P**GW**NVI**RER		
5141	SVVGWPTVRERMRRA					5141c	SV**P**GW**N**T**I**RER**I**RRA	5141q	**G**V**P**GW**N**T**I**RER**I**RRA
						5141d	SV**P**GW**NII**RER**I**RRA		
						5141h	SV**P**GW**NVI**RER**I**RRA		
5145	PAADGVGAVSRDLEK	5145a	PAADGVGA**A**S**Q**DL**A**K						
5149	GAITSSNTAANNADC	5149a	GAITSSNTAA**T**NADC	5149b	GAITSSNTAA**T**N**D**DC				
5151	AANNADCAWLEAQEE	5151a	AA**T**NADCAWLEA**H**EE	5151b	AA**T**N**D**DCAWLEA**H**EE				
5152	ADCAWLEAQEEEEVG	5152a	ADCAWLEA**H**EEEEVG	5152b	**D**DCAWLEA**H**EEEEVG				
5153	WLEAQEEEEVGFPVR					5153c	WLEAQEEEEVGFPV**K**		
5156	PVRPQVPLRPMTYKA					5156c	PV**K**PQVPLRPMTYK**G**		
5157	QVPLRPMTYKAAVDL					5157c	QVPLRPMTYK**G**A**L**DL		
5158	RPMTYKAAVDLSHFL	5158a	RPMTYK**G**AVDLSHFL						
5159	YKAAVDLSHFLKEKG	5159a	YK**G**AVDLSHFLKE**E**G			5159c	YK**G**A**L**DLSHFLKEKG		
5160	VDLSHFLKEKGGLEG	5160a	VDLSHFLKE**E**GGLEG						
5161	HFLKEKGGLEGLIYS	5161a	HFLKE**E**GGLEGL**VW**S						
5162	EKGGLEGLIYSQKRQ					5162c	EKGGLEGLIYSQ**Q**RQ		
5167	WVYHTQGYFPDWQNY	5167 *	WVYHTQGYFPDWQNY			5167 *	WVYHTQGYFPDWQNY	5167q	WVYHT**E**GYFPDWQNY
5169	FPDWQNYTPGPGIRY	5169a	FPDWQNYTPGPG**V**RY						
5170	QNYTPGPGIRYPLTF	5170a	QNYTPGPG**V**RYPLTF						
5171	PGPGIRYPLTFGWCF	5171a	PGPG**V**RYPLTFGWCF	5171b	PGPG**V**RYPLTFGWC**Y**				
5172	IRYPLTFGWCFKLVP	5172a	**V**RYPLTFGWCFKLVP	5172b	**V**RYPLTFGWC**Y**KLVP				
5173	LTFGWCFKLVPVEPE	5173a	LTFGWCFKLVP**M**E**ED**	5173b	LTFGWC**Y**KLVP**M**E**ED**				
5178	NEGENNSLLHPMSLH	5178a	N**A**GENNSLLHP**ICQ**H						
5179	NNSLLHPMSLHGMDD	5179a	NNSLLHP**ICQ**HG**I**DD						

* shows that the consensus and the autologous peptides had similar a. a. sequences, and a.a differences between consensus and autologous sequences are shown in bold.

Thus, using consensus B Nef peptides, the majority of CD8^+^ IFN-γ T-cell responses are not detected during either the primary or the chronic phase of HIV infection making it difficult to precisely define the role of functional CD8 T cell responses in driving virus polymorphisms and protection. A large number of CD8^+^ T-cell responses targeted autologous Nef peptides with no cross-recognition of consensus Nef peptides (Figure 2e). During the primary phase (day 92), of the 21 peptides recognized by CD8^+^ T cells in the high viremic subject, 20 (95%) of responses were detected with autologous HIV-1 Nef peptides compared to 16 (76%) responses detectable by the clade-B consensus Nef peptides. Similarly, for the low viremic subject only a portion (2/3) of the autologous peptides that stimulated early CD8^+^ T cell IFN-γ responses were detectable by clade-B consensus Nef peptides (day 155). This difference increased during the chronic phase, where only 5 out of 10 (50%) and 4 out of 13 (31%) autologous HIV-1 Nef responses were detectable by consensus clade-B Nef peptides for both high (day 448) and low (day 372) viremic subjects, respectively.

Only one of the early CD8^+^ T-cell IFN-γ responses in the high viremic subject was induced by the consensus peptide 5173 (LTFGWCFKLVPVEPE), which showed a very low response to one of the autologous variant peptides 5173a (LTFGWCFKLVPMEED) early in the infection. Interestingly, in the sample from the chronic phase of infection (day 448), the CD8^+^ T-cell IFN-γ response to this consensus peptide decreased and the magnitude of response to both autologous peptides 5173a and 5173b (LTFGWCFKLVPMEED and LTFGWCYKLVPMEED) remains stable or became strongly positive respectively. In the consensus sequence the predicted TV9 epitope (TFGWCFKLV) restricted by HLA-A*2301 contains a phenylalanine at position 6 which is a putative TCR contact residue, whereas the autologous sequence (TFGWCYKLV) contains a tyrosine at this position. Thus, as shown in Figure 2f, IFN-γ secreting CD8^+^ T-cells for autologous versus consensus peptide exhibited divergent patterns of evolution. The responses to autologous and consensus clade-B HIV-1 Nef peptides in the two subjects was then compared across time points (Table 2). There was only one case in which an increased response to the consensus peptide was noted in the absence of a concurrent increased response to the autologous peptide. Specifically, a pre-existing weak response to the consensus peptide 5151 (AANNADCAWLEAQEE) in the high viremic subject increased while the response to the autologous peptide 5151a (AATNADCAWLEAHEE) faded over time. For this peptide, the autologous peptide induced a higher IFN-γ response than the consensus peptide during the primary phase (day 92). This indicates that some ongoing CD8^+^ T cell responses that selected variant sequences in early infection no longer recognized viral variants during transition to chronic infection (day 448) could cross-react with consensus peptides leading to a false positive result of actual in vivo responses. Accordingly, all the CD8 T-cell IFN-γ responses that were exclusively induced by the consensus clade-B Nef decreased and no response was exclusively induced by the consensus peptides in the chronic phase (day 448). Most importantly, new responses induced by autologous mutated virus sequences were not detected using the consensus peptides. These results underscore the limitations in using consensus sequences to follow CD8^+^ T cell responses longitudinally that affect virus sequence evolution.

**Table 2:** Comparison of the trend of increase or decrease of CD8^+^ IFN-γ^+^ response to autologous and clade-B consensus HIV-1 Nef peptides in the two subjects.

	High Viremic (#039)		Low Viremic (#016)	
	Decreasing*	Increasing*	Decreasing*	Increasing*
No. of Consensus peptides	4	1	2	0
No. of Autologous peptides	2	4	0	9
No. of Both peptides	15	5	1	4
	*P = 0.1302* $		*P = 0.0040* $	

All responses ≥0.01% are taken into account.

* IFN-γ responses of CD8^+^ T-cells from primary to chronic phases.

$ Chi-square test.

In general, early in the primary infection, the total magnitude of IFN-γ responses to autologous 15-mer Nef peptides in the low viremic subject (day 155) was lower than that in the high viremic subject (day 92) (2. 8×10^2^ versus 9. 9×10^3^ in 10^5^ CD8 T-cells). A similar trend was observed during the primary phase for responses to the consensus 15-mer peptides, despite the two subjects having a similar magnitude of IFN-γ responses specific for the total viral proteome shortly after infection. The magnitude of Nef specific responses in the chronic infection did not differ significantly between the subjects (5. 3×10^2^ and 2. 4×10^3^ in 10^5^ CD8 T-cells, Mann Whitney, *P = 0.326*, respectively). While the majority of the CD8^+^ T-cell IFN-γ responses in the high viremic subject in the primary infection declined or disappeared over time, these responses increased from the primary (day 155) to the chronic phase (day 372) in the low viremic subject (Table 2). This gain of CD8 T-cell IFN-γ responses to autologous HIV-1 Nef peptides was consistent with an increase in the proteome-wide HIV-specific IFN-γ response seen in the low viremic subject from the early (day 155) to the chronic phase (day 372) time points detected in an ELISPOT assay (data not shown).

### Autologous HIV-1 Nef peptides induce a high frequency of polyfunctional IFN-γ producing CD8^+^ T-cell responses

Long term non progressors (LTNP) with protective HLA alleles show Gag and Nef-specific polyfunctional CD8 T cell responses in terms of Gr-B, Perforin, IFN-γ, MIP-1β, TNF-α, and IL-2 production [24]–[28]. Therefore, we next compared functional profiles of CD8^+^ T-cell subsets stimulated by autologous and consensus HIV-1 Nef peptides. The response to consensus peptide sequences at both time points for both study subjects were combined and compared to the results obtained from the responses to autologous peptides (Figure S3).

**Figure 3:**
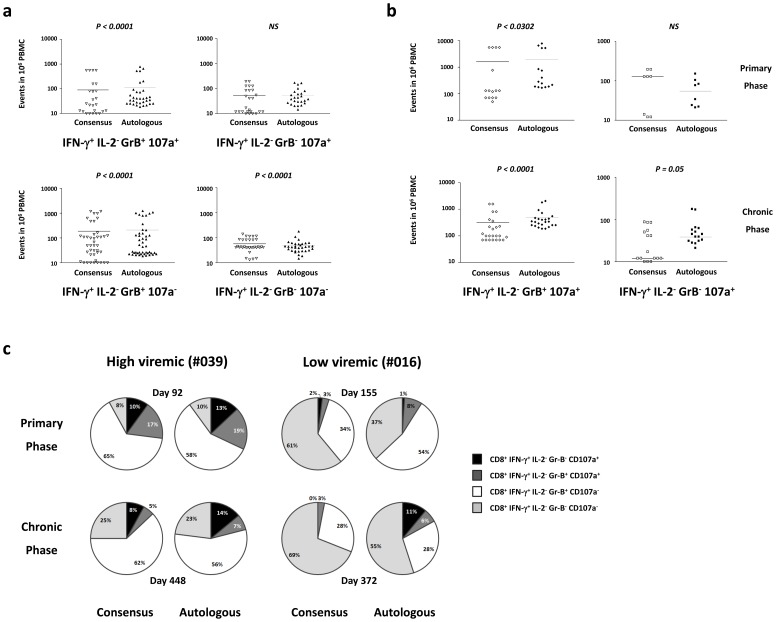
Greater frequency of polyfunctional IFN-γ producing CD8^+^ T-cells with autologous HIV-1 Nef peptides. (a) The frequency of CD8^+^ IFN-γ^+^ Gr-B^+^ CD107a^+^ detected by autologous HIV-1 Nef peptide and clade-B consensus Nef peptide stimulation. Functional subpopulations were defined by sequential gating of Gr-B and CD107a on CD3^+^ CD8^+^ IFN-γ^+^ cells. Each point in the graphs represents the response to one overlapping peptide. The responses of both subjects in both time points are shown together in each graph. (b) CD8^+^ IFN-γ^+^ Gr-B^+^ CD107a^+^ and CD8^+^ IFN-γ^+^ Gr-B^−^ CD107a^+^ T-cells detected by using autologous HIV-1 Nef and clade-B consensus Nef peptides in the chronic phase of infection. Each point in the graphs represents the response to one overlapping peptide. The responses of both subjects are represented in each graph. (c) Percentage of mono-, oligo- and polyfunctional CD8^+^ T-cells targeting pairs of consensus and autologous HIV-Nef peptides in an ICS assay. The graph represents an example of the difference in the function of stimulated CD8^+^ T-cells between the two subjects.

As shown in Figure 3a, there were more polyfunctional IFN-γ^+^ IL-2^−^ Gr-B^+^ CD107a^+^ CD8^+^ T-cells responding to autologous compared to consensus clade-B Nef peptides (*P<0.0001*, Wilcoxon signed rank). While we did not detect any significant difference between the IFN-γ^+^ IL-2^−^ Gr-B^−^ CD107a^+^ CD8^+^ T-cells, stimulated by either set of peptides as a whole, a higher frequency of IFN-γ^+^ IL-2^−^ Gr-B^−^ CD107a^+^ CD8^+^ T-cells responding to autologous Nef peptides in the chronic phase was observed (*P = 0.05*, Wilcoxon signed rank), but not in the primary phase (*P = 0.31*, Wilcoxon signed rank) (Figure 3b). Representative pie charts of mono-, oligo- and polyfunctional CD8^+^ T cells targeting a pair of consensus and autologous HIV-Nef peptides in an ICS assay for both subjects are shown in Figure 3c. Comparison of the functional subpopulations in the two subjects revealed that in the low viremic subject all polyfunctional subpopulations (IFN-γ^+^ IL-2^−^ Gr-B^+^ CD107a^+^, IFN-γ^+^ IL-2^−^ Gr-B^−^ CD107a^+^, IFN-γ^+^ IL-2^−^ Gr-B^+^ CD107a^−^) had a greater response to autologous rather than consensus HIV-1 Nef. A non-significant trend towards both autologous and consensus HIV-1 Nef peptides inducing CD8^+^ IFN-γ^+^ IL-2^−^ Gr-B^+^ CD107a^+^ responses in the high viremic subject was also noted (data not shown). Thus, polyfunctional responses with low magnitude IFN-γ production are more likely to be missed using consensus peptides as stimuli. It is noteworthy that responses to autologous peptide in the low viremic subject were predominantly single positive IFN-γ producing cells. In contrast, the overall frequency of responses in the high viremic subject were associated with Gr-B^+^ and CD107a^+^ cytotoxic effector responses compared to the low viremic subject during primary (90%versus 63%) and chronic infection (77% versus 35%) (Figure 3c).

### Higher frequencies of terminally differentiated IFN-γ producing CD8^+^ T-cells respond to the autologous Nef peptides

**Figure 4:**
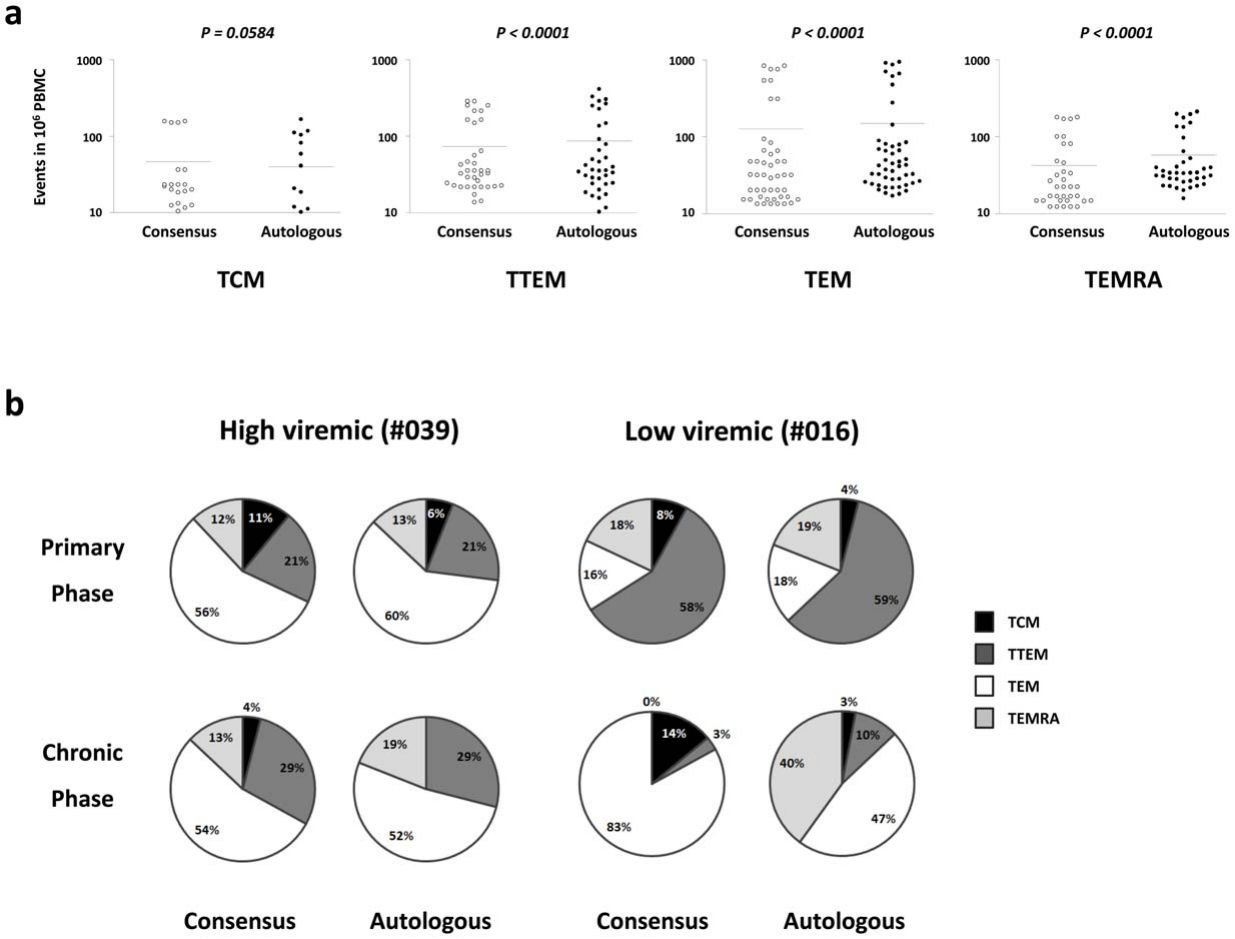
Greater frequency of terminally differentiated IFN-γ producing CD8^+^ T-cells responding to autologous HIV-1 Nef peptides. (a) The frequency of terminally differentiated effector (CD8^+^ IFN-γ^+^ CD45RA^+^ CCR7^−^ CD27^−^) and effector memory (CD8^+^ IFN-γ^+^ CD45RA^−^ CCR7^−^ CD27^−^) T-cells targeting autologous HIV-1 Nef and clade-B consensus HIV-1 Nef peptides in both subjects. The phenotypes of IFN-γ producing cells were detected by sequential gating of CD45RA, CCR7 and CD27 on CD3^+^ CD8^+^ IFN-γ^+^ T-cells. Each point in the graphs represents the response to one overlapping peptide. The responses of both subjects in both time points are represented in each graph. (b) The percentage of central memory (TCM), transitional memory (TTEM), effector memory (EM) and terminally differentiated effector (TEMRA) CD8^+^ T-cells targeting pairs of consensus and autologous HIV-Nef peptides in an ICS assay. The graph represents an example of the difference in the phenotype of stimulated CD8^+^ T-cells between the two subjects.

A comparison of the phenotype of IFN-γ producing CD8^+^ T-cells revealed a significantly higher frequency of terminally differentiated effector (CD8^+^ IFN-γ^+^ CD45RA^+^ CCR7^−^ CD27^−^) T-cells recognizing autologous versus clade-B consensus HIV-1 Nef peptides in both subjects (*P<0.0001*, Wilcoxon signed rank) (Figures 4a and 4b). Moreover, the frequency of effector memory (CD8^+^ IFN-γ^+^ CD45RA^−^ CCR7^−^ CD27^−^) T-cells was also higher in response to autologous HIV-1 Nef peptides (*P<0.0001*, Wilcoxon signed rank).

## Discussion

In our study, the magnitude of CD8^+^ T-cell IFN-γ responses to consensus viral sequences was found to underestimate the response to the autologous virus. Our results are consistent with a previous report [16], that showed a higher breadth and magnitude of CD8 T-cell responses against peptides from p24 Gag, Tat, and Vpr proteins of HIV, and a more recent report that showed of the 203 consensus and most common variant 10-mer peptides spanning Nef, 91 of these were recognized using consensus peptides and 46 additional regions were recognized using variants peptides only [29]. Interestingly, using web-based approach to CTL epitope, we found that for several peptides in both high viremic and low viremic subjects each peptide could be restricted by several HLAs. In case of escape mutation, a shift in the HLA restriction could also be observed (Doroudchi *et al*, manuscript in preparation). In addition, the majority of responses to autologous HIV-1 Nef showed higher frequencies of polyfunctionality and superior CD8^+^ T-cell maturation. These findings imply that subtle differences in consensus and autologous sequences, which may change the processing of the autologous antigen and the presentation of epitopes, can affect the breadth, magnitude, function and phenotype of the cognate CD8^+^ T-cell response ex vivo [30]–[32].

Consensus HIV sequences have been designed based on the most common a. a. in each position of a specific protein among circulating strains of HIV [33], [34]. Consensus peptides aim to overcome the high variability of HIV virus and to benefit from the cross-reactive properties of the epitopes [13]. The question, however, is to what extent a “cross-reactive” epitope in a peptide or protein can mimic the same response of the “true” epitope and more importantly how can CD8 T cell responses to cross-reactive peptides influence virus control? [35], [36]. The relative immunodominance pattern of epitopes restricted by HLA class I haplotypes expressed by an individual is one factor that shapes the breadth and hierarchy of CD8^+^ T-cell responses and depends on the sequence of an epitope and its MHC restriction [37]. It has further been shown that the functional avidity of CD8^+^ T-cell response to an epitope correlates with immunodominance of that epitope [25], [38], i. e. the more avid T-cell responses induce stronger signals and correlate with higher magnitude of CD8^+^ T-cell responses [39]. As less dominant epitopes map to the most variable parts of the protein, it is not unexpected that a set of cross-reactive epitopes in an artificially designed consensus HIV sequence fail to stimulate the same number or the same magnitude of functional responses in CD8^+^ T-cells [40]. Interestingly, in our study the magnitude of CD8^+^ T-cell IFN-γ responses to autologous peptides was almost two times greater than the responses to the consensus clade-B Nef peptides for both subjects (Figures 2a and 2b).

Since a large number of CD8^+^ T-cell IFN-γ responses targeted autologous Nef peptides with no cross-recognition of consensus Nef peptides (Figure 2e), a major part of CD8^+^ IFN-γ T-cell responses remained undetected by consensus peptides. The higher sensitivity of detection for autologous than consensus Nef peptides (i. e. the number of responses detected by each set of peptides) increased from more than 20% in the primary phase of infection to more than 50% in the chronic phase for both individuals. Moreover, a much higher specificity of responses (i. e. responses exclusively detected by the autologous or consensus peptides) was detected for autologous versus consensus Nef peptides in both the primary and chronic phases of infection (Figure 2e). These results indicate the sequence-specificity of the breadth and magnitude of the CD8^+^ T-cell responses to Nef epitopes and is consistent with previous reports showing that approximately one third of the responses to HIV proteins are not detectable by using HIV-1 clade-B consensus sequences [16]. Specific to our study, the absence of a response by consensus peptides in the chronic phase of infection and the higher percentage of increased CD8^+^ T-cell IFN-γ responses in response to autologous HIV-Nef peptides, emphasize that responses to viral polymorphisms driven by immune pressure may be missed by the consensus peptides. In addition, the CD8^+^ T-cell IFN-γ responses over time were found to differ between the consensus and autologous Nef peptides of the same region. This shows that the hierarchy of appearance of these responses is also dependent on subtle differences in the a. a. sequences of the peptides, which affect the sequence or processing of their inherent epitopes. Moreover, previous reports have shown the ability of the immune system to mount new CTL responses after epitope escape mutation during the chronic phase of infection [41], [42].

By comparing the function of CD8^+^ T-cell IFN-γ responses targeting autologous or consensus peptides, our study suggests that the quality of functional responses to HIV-1 Nef is also sequence specific. The difference was more obvious for the low magnitude responses, which were greater in the chronic phase of infection. The gains cannot be solely attributed to the mutations in autologous Nef protein, as the majority of these responses appeared in the low viremic subject in whom several original quasispecies evolved to one final dominant variant over time. Moreover, there was no significant increase in the a. a. divergence of the original and mutated quasispecies and the consensus sequences. Several studies have implicated subdominant CD8^+^ T-cell responses to HIV epitopes in the control HIV viral load [43], [44]. The advantage of autologous HIV-1 Nef peptides in detection of functional subpopulations in subdominant responses is, therefore, noteworthy. However, it is important to note that the CD8 immune responses against HIV were analyzed in this study using Nef consensus B and autologous 15-mers that overlap by 11 amino acids. Interestingly, despite that this method may not allow the identification of the minimal epitope (generally 9 or 10 amino acids in length) recognized by specific CTLs, our prediction analysis revealed broader CD8 immune responses against HLA class I minimal epitopes from autologous virus when compared to responses against minimal epitopes from consensus B peptides. These functional differences observed could be explained by amino acid changes within and flanking CD8 T-cell epitopes that impact the processing of the epitopes as shown in Table S2. It is also likely that optimal epitopes within the autologous 15-mer sequences are located in more favorable positions, which increase the binding avidity to HLA class I compared to consensus peptides. As a result, qualitatively different TCR signals in Nef-specific CTLs may be induced and cause differential activation of the T cells. In separate work, we defined the optimal 9-mer and 10-mer epitopes within the autologous 15-mer peptides in the context of escape mutation for both subjects (Doroudchi *et al*, manuscript in preparation).

The higher number of terminally differentiated effector CD8^+^ T-cells targeting autologous versus consensus HIV-Nef peptides points to the better maturation of CD8^+^ T-cells induced by autologous HIV-1 Nef peptides. Despite the cross-reactive nature of all TCR receptors, the TCR recognition of peptide-HLA is a very specific interaction, which provides the necessary signals for T-cell stimulation, maturation, and function. It has been shown that an a. a. difference in the cross-reactive SL9 Gag epitopes, restricted by HLA-A2, does not prevent the original interaction of peptide-HLA with TCR, however it affects the structure and stability of the final complex between peptide-HLA and TCR [45]. Although not addressed here, one could expect a difference in the strength of TCR signals provided by a “true” or “cross-reactive” epitope, and consequently a difference in functional and maturational outcome. The a. a. sequence divergence between the autologous and consensus peptides in our study ranged between 15% and 17% of the total Nef amino acid. Considering the small size of Nef protein (206 a. a.), this variation is substantial. The significant difference in the maturation state and polyfunctionality of responses induced by the autologous HIV-1 Nef and clade-B consensus peptides is, therefore, not unexpected. Several reports have shown that the extent and level of TCR engagement; i. e. antigen dose and ligand avidity, can affect the effector function of CD8^+^ T-cells [46], [47] and may result in partial activation and distinct patterns of differentiation to effector T-cells [48]. In our study, a bioinformatics approach was used to determine that both anchor and TCR contact residues responsible for the recognition of autologous epitopes by consensus clade-B peptides in both subjects (Table S2).

We showed that a majority of CD8^+^ T-cell responses to HIV-Nef are missed by using clade consensus peptides, particularly in chronic phase of infection. In addition, a more polyfunctional and better maturated CD8^+^ T-cell response to autologous HIV-1 Nef peptides was observed in both subjects, suggesting the inability of “cross-recognized” responses to mimic the “true” response.

Despite the low number of subjects recruited to this study, the large number of Nef protein responses investigated at two time points provides additional insight, to other studies which looked at only a few consensus or autologous peptides. A comprehensive analysis of Nef reactivity in these two subjects was chosen based upon the high epitope density and immune-reactivity to Nef that could reveal differences between autologous versus consensus Nef-specific responses [49], [50]. A key finding of this study is the functional importance of HIV sequence variation to HLA-peptide-TCR interactions mediating T-cell reactivity in immunoassays. It is unknown if Nef-specific responses had any influence on virus control since differences in the number of epitopes targeted in Gag could explain the ability to control virus replication between the two HLA-B*57/5801 subjects [51]. Since the ability to drive selective pressure positively correlates with the effectiveness of CD8 T cell responses in Gag to control virus load, we used bioinformatic tools to identify epitopes in regions with high sequence variability between consensus and autologous sequences that might identify protective responses in Nef (Table S2) [52]. Interestingly, we found that predicted epitopes in the NH2 termini of Nef showed the greatest variability in the low viremic subject yet this region is not frequently targeted and lacks structural -functional domains [3], [53]. Furthermore, we did not detect differential targeting of the immunodominant HW9 epitope in the HLA-B*5701 subject between autologous and consensus peptides, and recent data suggests that accumulation of A83G mutations within this epitope might represent evolution toward a new consensus sequence that has adapted to immune pressure.

In conclusion, our results strongly support the approach of using autologous peptides for immune-monitoring and the identification of CD8^+^ T-cell responses that target epitopes under strong selective pressure and that are most relevant for vaccine design.

## Supporting Information

Figure S1 Alignment of autologous virus sequences of Nef from the study subjects to HIV-1 clade-B consensus sequence. The amino acid sequence of the autologous virus determined in primary and chronic HIV-1 infection is shown for the two study subjects and aligned to the HIV-1 clade-B consensus sequence of Nef. An insertion of two a. a. (23-KA) and deletion of one a. a. (8-S) in the high viremic subject (#039) and a duplication of 9 a. a. (26-ERIRRAEPA) in low viremic (#016) subject were detected.
(TIF)

Figure S2 IFN-γ production in the high viremic subject. CD8 expression level versus the magnitude of IFN-γ production using PBMC for clade-B consensus and autologous HIV-1 Nef sequences, as a percentage of total CD8^+^ T-cells. The negative control = non-stimulated, Positive control tube = (SEB) used *Staphylococcus aureus* Enterotoxin B superantigen as stimulant.
(TIF)

Figure S3 Gr-B/CD107a production in the high viremic subject. Intracellular expression of Granzyme B versus the level of immobilized CD107a on the cell surface of IFN-γ^+^ CD8^+^ T-cells following stimulation with clade-B consensus and autologous HIV-1 Nef peptides. Functional subpopulation (%) in the IFN-γ^+^ CD8^+^ T-cell population is also shown.
(TIF)

Table S1 A summary of the viral load and T-cell counts in different time points for the two study subjects.
(XLSX)

Table S2 Predictions of proteasomal processing, TAP transport and peptide binding to HLA class I molecules.
(XLSX)
