# Supplementary figures and images for: Autologous HIV-1 Clade-B Nef Peptides Elicit Increased Frequency, Breadth and Function of CD8+ T-Cells Compared to Consensus Peptides
Source: PLoS One. 2012 Nov 19;7(11):e49562. doi: 10.1371/journal.pone.0049562 (PMC3501503; doi:10.1371/journal.pone.0049562)

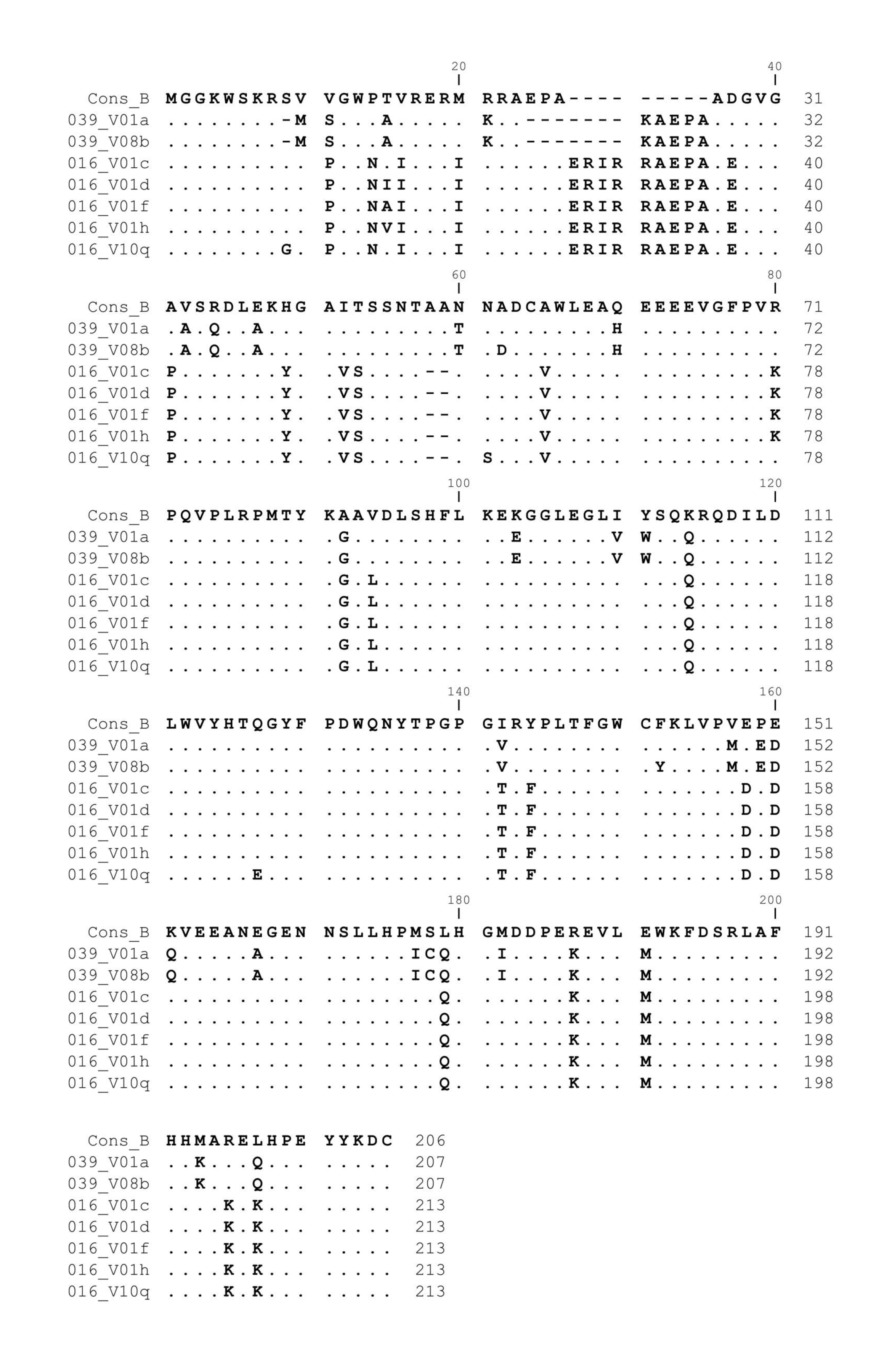

Supplement: Figure S1 — Alignment of autologous virus sequences of Nef from the study subjects to HIV-1 clade-B consensus sequence. The amino acid sequence of the autologous virus determined in primary and chronic HIV-1 infection is shown for the two study subjects and aligned to the HIV-1 clade-B consensus sequence of Nef. An insertion of two a. a. (23-KA) and deletion of one a. a. (8-S) in the high viremic subject (#039) and a duplication of 9 a. a. (26-ERIRRAEPA) in low viremic (#016) subject were detected. (TIF) [file pone.0049562.s001.tif]

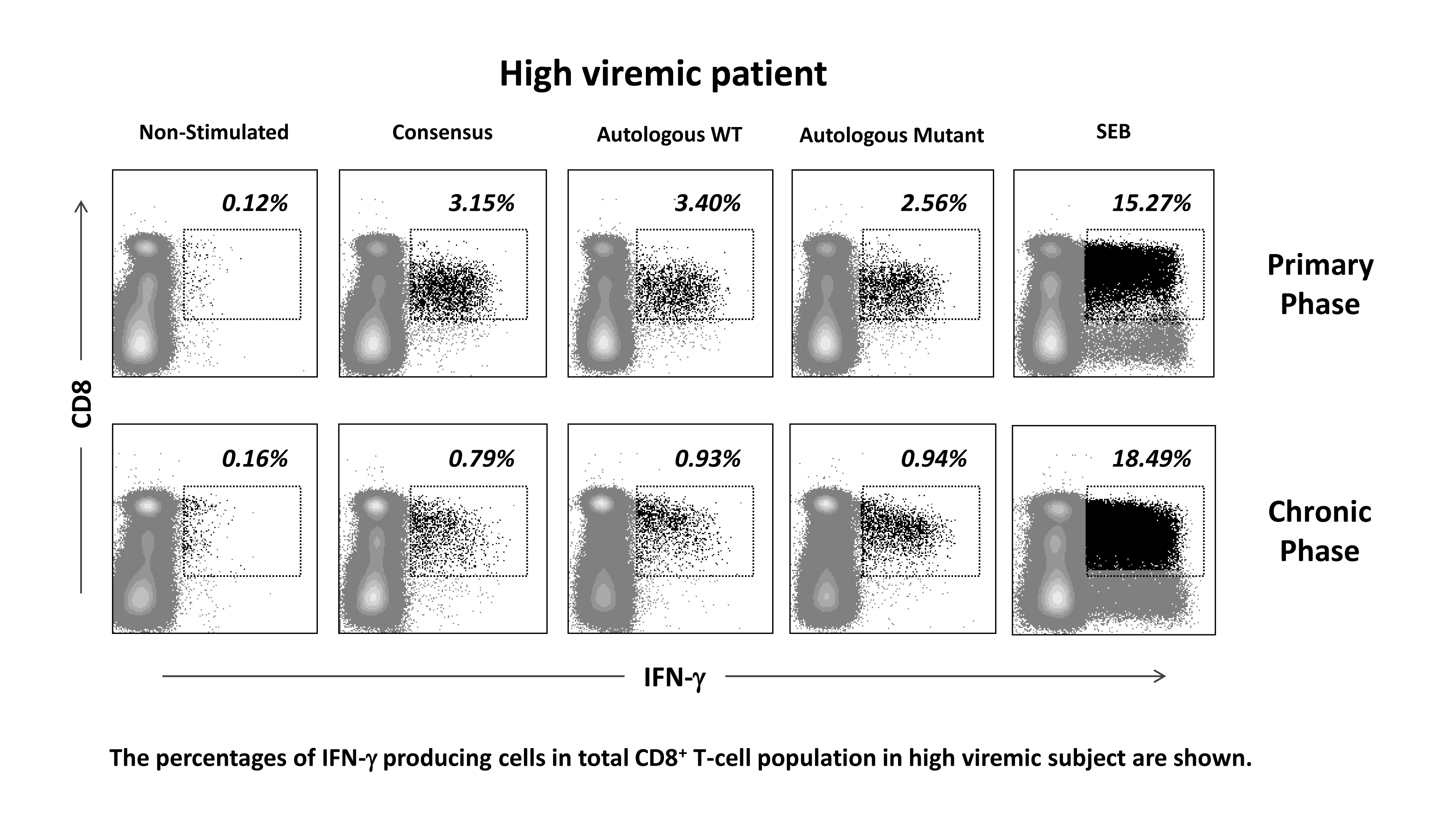

Supplement: Figure S2 — IFN-γ production in the high viremic subject. CD8 expression level versus the magnitude of IFN-γ production using PBMC for clade-B consensus and autologous HIV-1 Nef sequences, as a percentage of total CD8+ T-cells. The negative control = non-stimulated, Positive control tube = (SEB) used Staphylococcus aureus Enterotoxin B superantigen as stimulant. (TIF) [file pone.0049562.s002.tif]

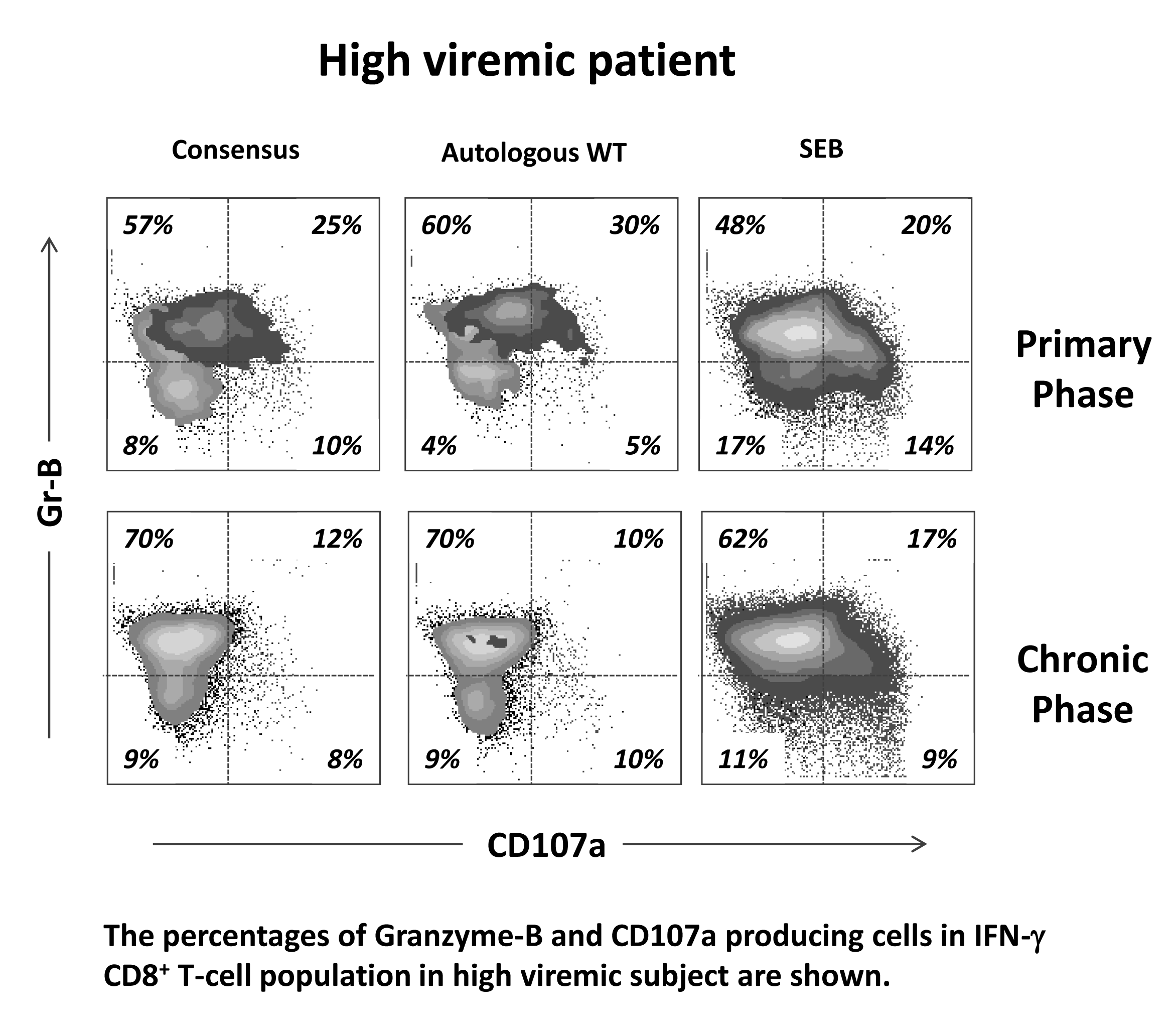

Supplement: Figure S3 — Gr-B/CD107a production in the high viremic subject. Intracellular expression of Granzyme B versus the level of immobilized CD107a on the cell surface of IFN-γ+ CD8+ T-cells following stimulation with clade-B consensus and autologous HIV-1 Nef peptides. Functional subpopulation (%) in the IFN-γ+ CD8+ T-cell population is also shown. (TIF) [file pone.0049562.s003.tif]
